# An Exploratory Study about the Characterization of Caffeine Consumption in a Portuguese Sample

**DOI:** 10.3390/bs12100386

**Published:** 2022-10-09

**Authors:** Patrícia Batista, João Peixoto, Patrícia Oliveira-Silva

**Affiliations:** Human Neurobehavioral Laboratory, Research Centre for Human Development, Universidade Católica Portuguesa, 4169-005 Porto, Portugal

**Keywords:** caffeine, caffeinated products, coffee, consumption, Portuguese

## Abstract

Caffeine is one of the most used psychoactive substances worldwide, with an impact in multiple spheres (individual, social, and economic). In addition, there is evidence of the physiological, cognitive, and emotional effects after consumption. This study aimed to examine caffeine consumption in a Portuguese sample by characterizing and understanding the pattern of consumption and the reasons for it. The sample was composed of 208 subjects recruited through the university’s social media channels to answer an online questionnaire between April and June 2020. The results showed a higher consumption in males and the group of subjects aged between 31 and 35 years. The coffee “express” is the most consumed source of caffeine in this Portuguese sample (70.2%). The data showed that improvement in alertness and the taste of products with caffeine were the main reasons for consuming caffeinated products. In conclusion, this study calls attention to the characterization of caffeine consumption to understand the need for such consumption and its effects on body functions and health. It is important to highlight the potential benefit of caffeine consumption due to its impact on the quality of life and health since this substance has effects not only on physical and mental health but also on social well-being.

## 1. Introduction

Caffeine (1,3,7-trimethylxanthine) is commonly accepted as one of the most used substances in the world [1,2,3]. In 2019, European Union Member States (EUMS) imported 3.0 million tons of coffee worth around €7.5 billion (there was a 14% increase in imports over 10 years) [4]. This substance, which is doubly classified as a food and as a stimulant drug, is present in seeds (i.e., cocoa beans or coffee beans), leaves (i.e., yerba matte), and fruits (i.e., guarana berries) of about 60 plant species [3,5]. However, caffeine can be found in many products such as coffee, sodas, energy drinks, tea, food (including chocolates), and even medication sold without a medic prescription [1,5,6,7].

Surveys carried out within the European Food Safety Authority (EFSA) Food Consumption Database revealed that the most consumed sources of caffeine are coffee, tea, caffeinated beverages (including energy drinks), and chocolate [8]. Furthermore, the data showed that coffee was the most consumed substance, contributing about 40–94% of total consumption. However, this consumption pattern is different in other countries, such as the U.K., where tea is the primary source of caffeine (accounting for 57% of global consumption) [9]. Specifically, in the case of energy drinks, their contribution to total caffeine consumption appears to be relatively low, ranging from 0% to 2.7% for children, 0–10.5% for adolescents, 0–4.2% for adults, and 0–1% for the elderly [8]. The fact that it is one of the most consumed substances globally is nourished both by the panoply of caffeinated products available and the varied motivations (related to the positive effects) mentioned by people to use it.

Increased performance, health benefits, taste, pleasure, habit, tradition, culture, and socialization are presented as factors that influence caffeine intake positively [10,11]. Additionally, the literature reports several vantages to health associated with caffeine consumption. There is scientific evidence that related caffeine consumption with reduced the risk of cardiovascular disease, type 2 diabetes, and some cancer types. It also potentially decreases the risk of developing neurodegenerative diseases (e.g., Parkinson’s and Alzheimer’s disease) [1,5]. Other authors have highlighted its role as a neuroprotective in patients with coinfected with human immunodeficiency virus and hepatitis C virus to reduce the risk of stroke, dementia, and depression in women [1].

However, negative impacts on health are also reported, such as osteoporosis, increased blood pressure, migraine, and an increased risk of developing anemia, gastritis, or stillbirths [1,5,12]. Additionally, it can lead to addiction, and increased self-reports of stress, anxiety, tension, nervousness, irritability, insomnia, and diuresis, especially when using higher dosages (>200 mg/occasion or >400 mg/day) [5,6,12].

In addition to its health benefits, caffeine is a substance with socializing effects and is dependent on the culture of a community. Coffee, the main source from which people ingest caffeine, is deeply linked to social utility and cultural associations [13,14]. Consequently, coffeehouses allow people to fulfill their needs for social interaction and belonging in the community [13]. This situation is confirmed when the reasons why people consume caffeine are explored [11,14,15].

So, this exploratory study aimed to characterize caffeine consumption in a Portuguese sample. It is important to know the amount of caffeine consumed in Portugal. It is also essential to understand the type of caffeinated products consumed and the reasons that lead to this consumption. In this study, we further intend to identify sex differences in the consumption of this methylxanthine and investigate discrepancies in caffeine intake between different age groups related to quantities and types of products consumed and the reasons for its consumption.

## 2. Materials and Methods

### 2.1. Participants and Study Design

This study used cross-sectional data from the Caffeine Consumption Questionnaire-Revised and the Caffeine Motives Questionnaire. An online survey was designed to conduct this study between 02 April and 19 June 2020.

The data were collected from a convenience sample of Portuguese people, including 208 subjects (*n* = 208) aged between 18 and 35 with Portuguese nationality, recruited through the university’s social media channels. The exclusion criteria were the presence of any health problem impeding caffeine consumption. Those criteria were chosen to ensure that participants could understand and sign the study consent form (i.e., older than 18 years old), but also that they were among the college-age young adults in order to align with other studies being conducted within the same research line.

This survey was conducted following the guidelines of the Helsinki Declaration, and all subjects signed written informed consent. Ethical approval was gained from the Ethics Lab of the Universidade Católica Portuguesa.

### 2.2. Instruments

The online questionnaire incorporated two instruments reported in the literature: the Caffeine Consumption Questionnaire-Revised [16], used to assess subjects’ consumption pattern and type of products consumed, and the Caffeine Motives Questionnaire, used to evaluate the motives that lead to that consumption [17].

#### 2.2.1. Caffeine Consumption Questionnaire-Revised (CCQ-R)

The CCQ-R is an instrument that measures self-reported weekly caffeine intake and the amount and type of caffeinated product consumed (i.e., coffee, soft drink, tea, cocoa, chocolate, and over-the-counter drugs) [16]. The questionnaire has 60 items, and it is a more accessible and more appealing way to answer these questions because it uses pictures portraying usually consumed products, visual aids for the servings’ sizes, and pictures of those products that are widely commercialized.

#### 2.2.2. Caffeine Motives Questionnaire-Revised (CMQ)

The CMQ is an instrument with 21 items that measure the reasons for caffeine intake. The answers are given on a 5-point Likert-type scale, where one represents an inexistence of consumption (‘never’), and five represents continuous consumption (‘always’) [17].

### 2.3. Design Procedure

The process of translation and validation of the instruments was a four-phase procedure. Firstly, the original instruments’ authors were contacted and provided permission to validate and use the instruments. Secondly, the questionnaires were translated to Portuguese by two bilingual individuals (fluent both in English and Portuguese), and the Portuguese version was translated back to English by the other two bilinguals. Third, after comparing all the versions and comparing the inter-rater reliability steps, the final Portuguese version was tested by 20 healthy volunteers recruited from a university for a pilot study. Fourthly and finally, three specialists approved the final version, and the instruments were incorporated into the online questionnaire, which was administered using an online survey, Qualtrics TM software (Provo, UT, USA), which automatically saved the anonymized in a password-protected database. Some items presented in the original instrument version were culturally responsive conceptual-cultural adapted for the Portuguese reality.

In addition to the questionnaires’ questions, standard demographic characteristics (sex, age, educational attainment, etc.) were also collected.

Subjects for this study were recruited by social networks. Initially, they received an explanation about the study’s aims, the informed consent, as well as the guarantee of anonymity, and the possibility of withdrawing at any time without any harmful consequences. Then, those who intended to participate in the study received the researcher’s e-mail address to ensure they would be able to establish contact if needed.

### 2.4. Statistical Analysis

The data were first exported from Qualtrics into Microsoft Excel, where it was screened for missing information.

The software used for the statistical analysis was IBM SPSS® (Statistics for Windows, Version 25.0. Armonk, NY, USA).

Descriptive statistics were used to summarize subjects’ sociodemographic characteristics. Although the data were not normally distributed, the mean (*M*) was used as a measure of central tendency and standard deviation (*SD*) as a measure of dispersion to facilitate the reader’s understanding and comparison with results from other studies. Scale variables were tested for normality by carrying out Kolmogorov–Smirnov and Shapiro–Wilk tests. In the inter-subject analysis, the Mann–Whitney *U* test (given the non-normal distribution of data), the chi-square test (*χ*^2^) (due to the nature of the dependent variables), and the Kruskal–Wallis *H* test were used (Dunn’s multiple comparisons post hoc were also performed with the *p*-value adjusted using the Bonferroni correction).

## 3. Results

### 3.1. Characteristics of the Sample

Of 208 participants, most were female (*n* = 135, 64.9%), and aged 18-34 years old, with a mean age of 25 years old (*M* = 25.29, *SD* = 4.26). Participants were divided into four age groups to facilitate the analysis: (1) the 18–21 age group has 42 subjects (20.2%); (2) the 22–25 age group with 68 individuals (32.7%); (3) the 26–29 age group with 62 subjects (29.8%); and (4) the 31–35 years old.

Most of the participants reported being single (87%) and 75% with academic graduation. Regarding the professional situation, the sample is mainly divided between students (39.9%) and employed subjects (43.8%) (Table 1).

### 3.2. Caffeine Intake

**Figure 1:**
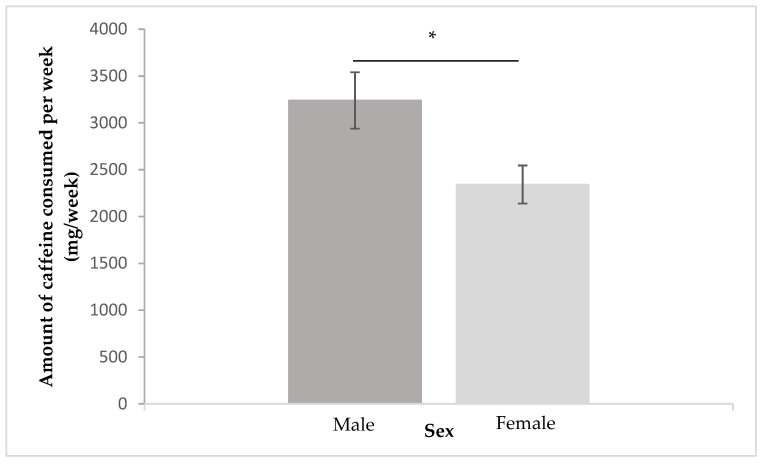
Caffeine intake per week (mg/week) by sex (* *p* < 0.05).

Participants reported an amount of caffeine consumed per week of 2656.71 mg/week. Considering sex, males reported to weekly consume more caffeine (M = 3238.65; SD = 2573.63 mg/week) than females (*M* = 2342.03; *SD* = 2361.28 mg/week). Statistically significant differences were found between male and female as shown (*U* = 3734, *z* = −2.88, *p* = 0.004) (Figure 1).

Also, the data showed that caffeine consumption is significantly higher in participants belonging to the age groups 26–29 and 30–35 (*M* = 3001.5, *SD* = 2669.61; *M* = 3630.69, *SD* = 2873.89, respectively). The youngest age group was the one who reported a lower consumption (*M* = 1651.64, *SD* = 1711.35).

**Figure 2:**
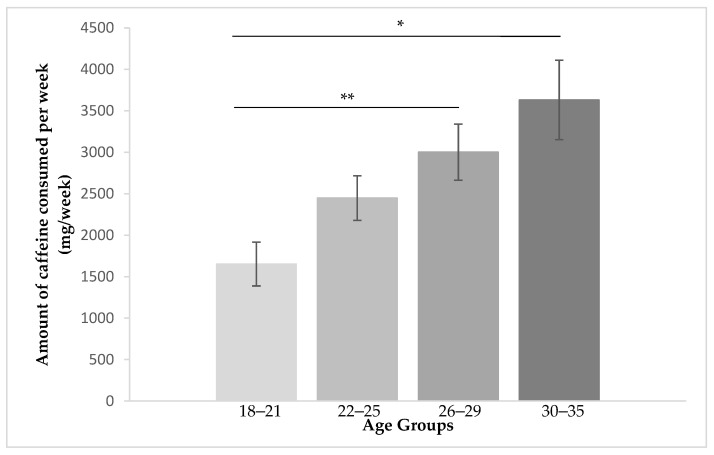
Caffeine intake per week (mg/week) by age groups (years) (* *p* < 0.05; ** *p* < 0.01).

A Kruskal–Wallis test revealed a statistically significant difference between the different age groups, *χ*^2^(3) = 15.1, *p* = 0.002, followed by the Dunn’s multiple comparisons post hoc test performed on all six pairs of groups, which confirmed the statistically significant difference in the average amount of caffeine consumption between younger individuals and 26–29 and 30–35 age groups (*p* = 0.012; *p* = 0.002, respectively) (Figure 2).

Considering coffee consumption by sex and age, it was found that older males (30–35 age group) reported consuming more caffeine than the other age groups (*M* = 4791.04, *SD* = 2856.97). Moreover, older females also had the highest consumption (*M* = 2892.29, *SD* = 2691.42) compared to the other age groups. In addition, statistically significant differences were found between the different age groups within each sex, as demonstrated by the Kruskal–Wallis *H* tests for males and females (*χ*^2^(3) = 8.02, *p* = 0.046; *χ*^2^(3) = 9.99, *p* = 0.019, respectively), followed again by Dunn’s multiple comparisons post hoc test including all six pairs of groups for each sex, showing a significant difference in the average amount of caffeine consumption between older males and the 22–25 age group (*p* = 0.029), and younger females (18–21) and the 26–29 age group (*p* = 0.026).

### 3.3. Caffeine Sources

The source of caffeine that had the highest reported consumption and, consequently, is the source from which most of the caffeine consumed comes is coffee (*M* = 2313.47, *SD* = 2266.31). However, the consumption is scarce if considering the other caffeine sources (Table 2). It is also interesting to highlight that, although food supplements were included in the questionnaire, the respondents gave any answer related to them.

### 3.4. Reasons for Caffeine Consumption

**Figure 3:**
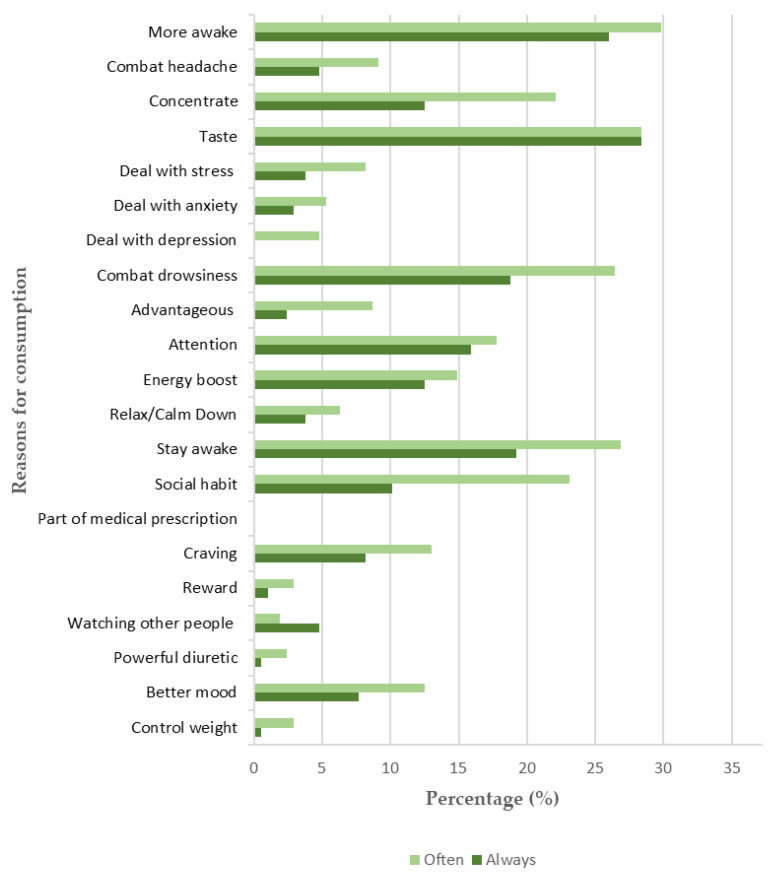
Reasons for caffeine consumption in a Portuguese sample.

Concerning the motives to consume caffeinated products, subjects reported that the two main reasons are the taste of caffeinated drinks and their physiological effect (mainly, to stay awake) (Figure 3). Based on the purpose of the study, only the most significant reasons reported by the subjects are presented.

According to the data, considering the reasons to which both sexes responded, ‘always’ or ‘often’, females significantly consume more caffeine than males to combat headaches (17.8% and 6.9%, respectively), as demonstrated by the Mann-Withney *U* test performed (*U* = 5907.5, *z* = 2.59, *p* = 0.01). On the other hand, males significantly consume more caffeine than females due to the taste of caffeinated beverages (74% and 47.4%, respectively), due to the energy boost they feel after the consumption (35.6% and 23%, respectively), and as a social habit (43.8% and 27.4%), as demonstrated by Mann-Witney *U* tests performed (*U* = 3246.5, *z* = −4.18, *p* = 0.000; *U* = 4054.5, *z* = −2.18, *p* = 0.029; *U* = 3585, *z* = −3.33, *p* = 0.001, respectively).

Considering age groups and the reasons why people reported consuming caffeine ‘often’ or ‘always’, older individuals (i.e., 30–35) pointed out social habits, caffeine’s ability to improve mood, and the rewarding action of this substance, as important reasons why they consume caffeine (50% and 33.4%, respectively) as demonstrated by the Kruskal–Wallis *H* tests performed (*χ*^2^(3) = 9.33, *p* = 0.025; *χ*^2^(3) = 8.87, *p* = 0.031, respectively).

### 3.5. Perceived Caffeine Effect

Most subjects recognized perceived positive/favorable/desired effects (70.7%). Even so, negative/adverse/unwanted effects were also reported (9.6%) or even the total absence of effect (19.7%). Although both sexes identified a positive effect, more females identified a negative effect compared to males (11.1% and 6.8%, respectively). Despite these results, no association was found between sex and the perceived caffeine effect, as demonstrated by Pearson’s chi-square test performed, *χ*^2^ (2, *N* = 208) = 2.09, *p* = 0.352.

Regarding age, all age groups reported a positive/desired effect, although younger subjects (18–21) more regularly identified the absence of an effect (31%). No association between age and the perceived caffeine effect was found, as revealed by the Pearson’s chi-square test performed, *χ*^2^ (6, *N* = 208) = 6.6, *p* = 0.36. It is interesting to note that all older males (30–35) reported a perceived caffeine effect as positive/desired. In addition, younger males did not report any negative/undesired effects. Older females were the group that most reported a negative/unwanted effect (18.2%). Pearson’s chi-square test showed no association between females’ age and the perceived caffeine effect, *χ*^2^ (2, *N* = 208) = 2.51, *p* = 0.286.

### 3.6. Subjective Sensitivity to Caffeine Effects

Related to subjective sensitivity to caffeine effects, the data showed that 63.5% of subjects reported that they do not perceive themselves as particularly sensitive to those effects. Considering sex, both males and females reported the same (69.9% and 60%, respectively), and there was no association between sex and the sensitivity to the effects of caffeine, as demonstrated by the Pearson’s chi-square test performed, *χ*^2^ (1, *N* = 208) = 1.99, *p* = 0.159. Concerning age, only older subjects (30–35 age group) reported perceiving themselves as more sensitive to the effects of caffeine. However, no association between age and sensitivity to the effects of this substance was found, as demonstrated by Pearson’s chi-square test performed, *χ*^2^ (3, *N* = 208) = 5.61, *p* = 0.132. The data also showed that older males (30–35) reported a more considerable subjective sensitivity to caffeine than the other age groups of the same sex (57.1%). The same was also found in females, although in a more balanced way (50%). The Pearson’s chi-square test performed showed no significant statistical association between females’ age and the perceived sensitivity to caffeine, *χ*^2^ (3, *N* = 208) = 2.95, *p* = 0.399.

## 4. Discussion

The present study characterized the caffeine consumption of caffeinated in a Portuguese sample, related to quantities and types of products consumed, the reasons for this consumption, and the perceived and subjective sensitivity to caffeine effects.

The data showed that males reported higher consumption compared to females, and these findings are in line with other studies, such as Pinhão and collaborators and Knapik and colleagues [18,19]. However, Dillon and colleagues’ (2019) study did not find results in line with ours, although their subjects had a much lower average age than in the present study [20]. Our results are also contrary to the ones found in Mahoney and collaborators [7]. In addition, it is notable that women reported more negative effects regarding consumption than men, which may condition the percentage of consumption to be higher in men.

Our data also showed that older subjects (30–35) consume more caffeine than other age groups, which is also in agreement with studies by Pinhão and collaborators, and Knapik and colleagues [18,19]. In the study conducted by Pinhão and collaborators, it was verified that the age group from 30 to 40 years old was the one that consumed the most caffeine, and in the second, individuals from 30 to 39 years old presented consumption similar to those ≥ 40 years old [18,19]. This consumption at older ages could be compromising because people at an older age rage also say they are more sensitive to consumption, possibly because they are consuming too much.

Concerning caffeine consumption, coffee remains the primary source of caffeine intake, which corroborates the results in most European countries and the United States of America [7,19,21,22]. Approximately two billion coffee “express” are estimated to be consumed every day in the world [23]. Other caffeine sources consumed are sodas, energy drinks, tea, chocolate drinks, food (i.e., chocolate, yogurt, ice cream, candies, and baked food), food supplements, and over-the-counter drugs (although some products had little expression in the present study) [1,5,6,7]. A comparative example in the literature is Verster and Koenig (2018), who summarized the caffeine intake of 275,000 children, adolescents, and adults and found that the major sources of caffeine reported were coffee and tea, followed by carbonated soft drinks [24]. However, the same authors did not make a significant contribution from other caffeinated beverages (e.g., energy drinks) or foods (e.g., chocolate) across all age groups [24]. These results showed that coffee (particularly espresso) and tea are key contributors to daily caffeine intake.

Caffeine consumption has been positively associated with age in several studies, and a moderately positive correlation with age was also found in the current one. The influence of caffeine consumption on well-being and cognitive function has been demonstrated in several studies involving humans. For instance, Repantis and colleagues found caffeine consumption positive effects on mood and processing speed, Sherman and collaborators found a positive effect on memory during the non-optimal times of day, as in the early morning and anxiety, Marczinski on vigor activity, as well as staying awake during the working hours reported in the Pegado and collaborators study [22,25,26,27]. One could also understand these data based on Fernandes and colleagues’ work, who argued that young coffee consumers show a more occasional pattern. In contrast, individuals aged over 30 are essentially everyday consumers [28].

When quantifying the caffeine consumption by week, the data were found to be 2656.71 mg/week (approximately 379.53 mg/day), which is higher than the amount consumed per day, for example, in New Zealand or the United States [2,7,19]. Unlike the values reported in mg/day in some studies, for instance, in Knapik and colleagues, and Stachyshyn and colleagues, we chose to present this result in mg/week to avoid the variability that occurs per day [2,19]. This consumption can be influenced, among other reasons, by the deep-rooted Portuguese culture [22]. So, in addition to the knowledge of caffeine consumption (in general), it is important to understand the reasons that lead people to consume it. Thus, the results showed that the two main reasons are the taste of caffeinated drinks and their physiological effect (i.e., to stay awake). These reasons for consuming caffeinated drinks are in line with the findings of other studies [11,14,22]. These results were similar for both sexes; however, other reasons were considered. For example, females reported using caffeine more often to combat headaches. At the same time, males seem to like the taste of caffeinated drinks more and use it more often as a social component, a result aligned with other studies [29,30]. The product availability and the Portuguese social habit of regularly consuming caffeinated products in different social contexts can contribute to the high-dosage consumption of this kind of product. So, this type of characterization study is important to know our sample well and its consumption since excessive consumption can condition physical and mental health. However, information regarding the health effects of caffeine consumption is controversial [23].

Regarding subjective sensitivity to caffeine, most subjects (considering the entire sample) reported not being especially sensitive to its effects, and the same was true for both sexes. Still, older subjects considered themselves more sensitive to the effects of this methylxanthine (although no association was found between age and sensitivity to the effects of caffeine). Both older males and older females were the groups that reported being more sensitive to the effects of caffeine when compared to the other age groups within each sex. When we approached the question related to the perceived effect of caffeine, we understood that, globally, the subjects considered that caffeine has a positive/desired effect on them. Considering the sex, and although the aforementioned is true for most subjects within each sex, we found that more females reported a negative/unwanted effect when compared to males, which is one of the reasons that may explain a lower consumption of caffeine by them (although no association was found between sex and perceived effect). Regarding age, we found that the younger subjects were the group that more regularly identified the absence of an effect. We also found that all older males identified a positive effect (a reason that may help explain the fact that this age group consumes more caffeine than other male age groups) and that no younger males identified any negative effects. Concerning females, the older ones were the ones who reported perceiving the effects of caffeine as negative/unwanted. The following results are contrary to the studies presented by Botella and Parra and Atwood and his collaborators, who suggested that women appear to be less sensitive to the caffeine’s effect and that high-dose consumers (>200 mg/day) are more likely to feel the positive effects of this substance and less likely to experience negative effects when compared to moderate-dose users (<200 mg/day) [31,32].

Analyzing the pattern of caffeine consumption in society is important not only to characterize the consumption of this psychoactive substance that has an impact on the physical and mental health of individuals but also on the performance of the individual as a social being since this substance is often consumed as a socializer. Thus, further studies should be designed to understand the effect of this substance on society.

## 5. Limitations 

This study presents some limitations that should be acknowledged, such as the selection of a cross-sectional study and online data collection during the lockdown (because of the COVID-19 pandemic situation), which does not allow further explanations if subjects have any questions/doubts, and could influence the perception of caffeine consumption. Another limitation was the sex disparity; our sample is mostly female, which made it impossible to generalize the results. The majority of the subjects in this study were young and women, which represents an obvious limitation in this study due to the poor sample representativeness across all sociodemographic variables.

## 6. Conclusions

In conclusion, the current findings suggest that coffee “express” is the main source of caffeine consumed and stands out as the main reasons for consumption, the taste of caffeinated drinks, and their physiological effect (awake).

The study of this substance and its characterization are therefore of great importance for research and public health since it allows the adoption of new practices or changes in current practices that contribute to better health. Understanding the quantities consumed, the most consumed products, and the reason why they are consumed will allow government entities and health and education organizations to develop health literacy and strategic health promotion plans.

The ample availability, the well-known cognitive and physiological effects, and the economic and social impacts associated with caffeinated products, particularly coffee, seem to be translated into a vast consumption of this substance in Portuguese society. However, further research is needed to understand variations between sexes in terms of caffeine consumption, the sources, the effects of timing, and the dose of caffeine consumption in this population.

## Figures and Tables

**Table 1:** Subjects’ sociodemographic characteristics.

Variable	*n* (%)
Total	208 (100)
**Sex**	
Male	73 (35.1)
Female	135 (64.9)
**Age Groups**	
18–21	42 (20.2)
22–25	68 (32.7)
26–29	62 (29.8)
30–35	36 (17.3)
**Marital Status**	
Single	181 (87)
Union	11 (5.3)
Married	16 (7.7)
**Education**	
Elementary/Primary Education (up to 9th grade)	2 (1)
Secondary Education (up to 12th grade)	50 (24)
Superior	156 (75)
Undergraduate Degree	81 (38.9)
Master’s Degree	74 (35.6)
Doctor Degree	1 (0.5)
**Work Status**	
Student	83 (39.9)
Employed	91 (43.8)
Unemployed	9 (4.3)
Student worker	25 (12)

**Table 2:** Sources and concentrations of caffeine consumption by sex and age group.

**Caffeine Sources**			**Sex**			
	**Male**		**Female**			
	*Mean* (*SD*)		*Mean* (*SD*)		**Test (*U*)**	***p***
All Sources	3238.65 (2573.63)		2342.03 (2361.28)		3734	0.004
Coffee	2993.7 (2502.82)		1945 (2044.52)		3597	0.001
Decaffeinated Coffee	4.04 (13.96)		5.44 (24.57)		4710.5	0.398
Energetic Drinks	6.58 (32.5)		-		-	-
Tea	97.23 (160.14)		132.78 (191.92)		5757	0.034
Food with Caffeine	67.34 (331.41)		208.13 (1190.87)		5794	0.023
**Caffeine Source**	**Age Groups (years)**					
	**18–21**	**22–25**	**26–29**	**30–35**		
	*Mean* (*SD*)	*Mean* (*SD*)	*Mean* (*SD*)	*Mean* (*SD*)	**Test (*H*)**	***p***
All Sources	1651.64 (1711.35)	2447.49 (2223.73)	3001.49 (2669.61)	3630.69 (2873.89)	15.1	0.002
Coffee	1424.07 (1722.25)	2113.81 (2034.2)	2531.73 (2226.27)	3352.34 (2849.84)	14.05	0.003
Decaffeinated Coffee	4.64 (22.75)	3.89 (17.81)	5.63 (25.74)	6.11 (18.48)	2.83	0.418
Energetic Drinks	-	3.53 (29.1)	0.97 (7.62)	5 (22.1)	-	-
Tea	63.42 (125.72)	131.73 (169.72)	130.96 (144.89)	146.71 (283.17)	8.24	0.041
Food with Caffeine	104.73 (306.85)	125.61 (402.22)	293.96 (1724.86)	51.35 (165.12)	2.66	0.447

## Data Availability

Not applicable.
